# Regulation of cancer by inflammasomes: from inflammation to tumorigenesis

**DOI:** 10.3389/fimmu.2025.1611719

**Published:** 2025-07-07

**Authors:** Shivani Malvankar, Pundrik Jaiswal, Panchami P. Bhat, Subhash Mehto

**Affiliations:** ^1^ Immunobiology Laboratory, Department of Biosciences and Bioengineering, Indian Institute of Technology, Dharwad, Karnataka, India; ^2^ Laboratory of Cellular and Developmental Biology, National Institute of Diabetes and Digestive and Kidney Diseases, The National Institutes of Health, Bethesda, MD, United States

**Keywords:** inflammasomes, radiation, cancer, NLRP3, NLRC4, AIM2

## Abstract

Inflammation is closely linked to the development and progression of cancer, as well as the effectiveness of cancer treatment. Inflammation is an immune response triggered when the immune system detects harmful stimuli such as pathogens, damaged cells, or toxic substances through pattern recognition receptors (PRRs). This activates signaling pathways and inflammasomes leading to the release of pro-inflammatory cytokines. In chronic inflammation, immune cells such as T and B lymphocytes, play a significant role in amplifying and sustaining the inflammatory response. The Inflammasomes are protein complexes that respond to microbes and danger signals, triggering an inflammatory response. Key inflammasomes, including NLRP3, AIM2, and NLRC4, regulate the release of proinflammatory cytokines and induce pyroptosis. While inflammasome activation is vital for immune defense, its dysregulation is associated with various diseases, including cancer. The relationship between inflammasomes and cancer is complex and varies depending on the context, with studies showing both promotion and inhibition of tumor growth. This review highlights the connection between microbes and radiation induced inflammatory regulators and cancer, stressing the need for research to understand the mechanisms through which inflammasomes and other inflammatory sensors control cancer.

## Introduction

Inflammasomes are multiprotein complexes that are assembled in response to PAMPs (pathogen-associated molecular patterns) and DAMPs (danger-associated molecular patterns), triggering an innate immune and inflammatory response (1–5). These complexes consist of sensor proteins, an adaptor protein, and caspase-1 (6). Among the most well-studied inflammasomes are the NLRP3 inflammasome, AIM2 inflammasome, and NLRC4 inflammasome (7–9). These inflammasomes sense microbes or cellular stress, recruit the adaptor protein ASC, and activate caspase-1, which cleaves precursor forms of IL-1β and IL-18 into their active forms, leading to inflammation and pyroptosis cell death (10).

**Figure 1:**
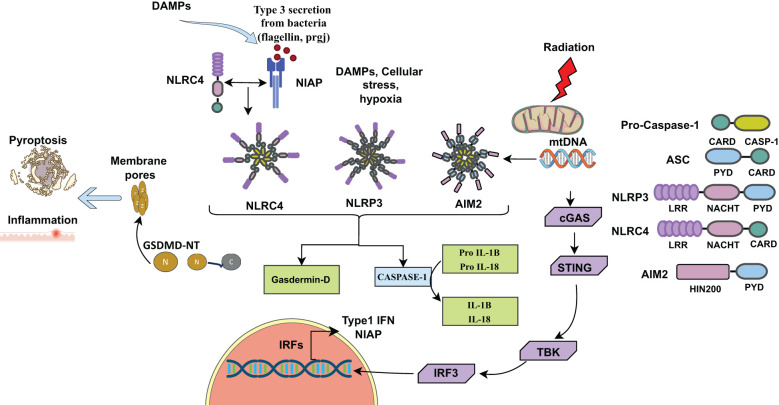
Inflammasomes activated by DAMPs, (ROS) released from dying cells due to cellular stress, infection, or radiation. Inflammasomes consist of sensors (NLRP3, NLRC4, AIM2), adaptors (ASC), and caspase. NLRP3, NLRC4, AIM2 along with ASC cleaves pro-caspase-1 into active caspase-1, which cleaves pro- IL-1β and pro-IL-18 into their active forms, IL-1β and IL-18. Active caspase-1 also cleaves Gasdermin D (GSDMD) into N-terminal (GSDMD-N) and C-terminal (GSDMD-C) fragments. GSDMD-N forms pores into the cell membrane for IL-1β and IL-18 secretion, initiating inflammation and pyroptotic cell death. Radiation-induced mitochondrial damage releases mtDNA or DSBs into the cytosol, activating the AIM2 inflammasome complex. Additionally, mtDNA activates the cGAS-STING pathway, leading to type I interferon (IFN-I) production. DAMPs (Damage-associated molecular patterns), DSBs (Double-stranded breaks).

Damage-associated molecular patterns (DAMPs), such as reactive oxygen species (ROS) are actively produced in radiation-induced tissue injury (11, 12). These reactive species activate inflammasomes, particularly the NLRP3 inflammasome, through K+ efflux via P2X7 channels (13–15). The efflux of potassium ions serves as a priming signal for NLRP3 activation (14, 15). Ionizing radiation also causes DNA damage, including double-strand break (16), which are recognized by the AIM2 inflammasome (**Figure 1**) (17–19).

The role of inflammasomes in cancer is notably dual-faceted, exhibiting both tumor-promoting and tumor suppressive functions depending on the context, cell type, and tumor microenvironment. On one hand, inflammasomes such as NLRP3 and AIM2 can suppress tumorigenesis by enhancing anti-tumor immunity through the activation of caspase-1 and subsequent maturation and release of pro-inflammatory cytokines like IL-1β and IL-18, which recruit and activate immune cells (20). On the other hand, chronic or dysregulated inflammasome activation can contribute to a pro-tumorigenic environment by sustaining inflammation, promoting angiogenesis, and supporting tumor cell proliferation and metastasis. For example, in colitis-associated colorectal cancer NLRP3 inflammasome activation has been shown to exert protective effects in some models, while in others, it contributes to tumor progression by enhancing the production of inflammatory mediators (21, 22). This dichotomy underscores the complexity of inflammasome signaling in cancer and highlights the need for context-specific therapeutic targeting.

Radiotherapy is a key treatment option for various types of tumors, including lung, breast, prostate, skin cancer, and renal cell carcinoma (23). However, radiation-induced tissue damage and inflammation can complicate its use in cancer therapy. Dysregulation of inflammasomes has been associated with development and progression of several inflammatory diseases and cancer. Targeting inflammasomes and their downstream signaling pathways may offer a promising therapeutic strategy for cancer treatment, either through suppressing the pro-inflammatory effects or enhancing their tumor-suppressive actions. Suppressing inflammasomes activation can reduce radiation-induced tumor damage, thereby potentially enhancing the overall efficacy of radiotherapy. Here, we discuss the implications of NLRP3, AIM2 and NLRC4 inflammasomes in both cancer development and tumor suppression as well as the potential for future investigations in this context.

## NLRP3 inflammasome activation and its role in cancer progression

The NLRP3 (NOD, LRR and pyrin domain containing protein 3) inflammasome is a multimeric protein complex that is assembled and activated upon recognition of molecular patterns (PAMPs or DAMPs) to induce the secretion of inflammatory cytokines IL-1β and IL-18 as part of innate immune responses. The NLRP3 inflammasome consists of NLRP3 proteins, which interact with ASC (apoptosis-associated speck-like protein containing a CARD). The NLRP3-ASC complex then recruits caspase-1, which cleaves pro- IL-1β and pro-IL-18 into IL-1β and IL-18 (24). Caspase-1 also cleaves gasdermin D (GSDMD) into a N-terminal fragment (GSDMD-N) and a C-terminal fragment (GSDMD-C). N-terminal fragment (GSDMD-N) forms pores in the membrane through which IL-1β and IL-18 are secreted from the cells (**Figure 1** **).** Aberrant activation of the NLRP3 inflammasome can lead to chronic inflammatory diseases and autoimmune disorders (25–29).

In addition to inflammatory responses, the NLRP3 inflammasome has been associated with various types of cancer exhibiting both pro-tumorigenic and anti-tumorigenic effects (**Table 1** **).** Ju et al. (2021) identified altered expression of NLRP3 inflammasome-related genes in 15 out of 24 cancer types studied (30). Elevated NLRP3 expression is elevated in several cancer types, such as colorectal carcinoma (CRC), oral squamous cell carcinoma (OSCC), and non-small cell lung cancer (NSCLC) (31–34). The activation of the NLRP3 inflammasome and its impact on tumorigenesis differ across different types of cancer. For instance, in CRC, NLRP3 inflammasome activation is associated with epithelial-mesenchymal transition (EMT) and contributes to cancer progression (35). In breast cancer, autocrine IL-1β secretion driven by the NLRP3 inflammasome promotes EMT, and metastasis in breast cancer (36). Laing et al. (2020) showed that lung cancer cells release exosomes containing TRIM59, an E3 ligase, which are transferred to macrophages, leading to NLRP3 inflammasome activation and promoting lung cancer progression (37). In OSCC, the NLRP3 inflammasome promotes proliferation, migration and invasion of cancer cells (32). In pancreatic ductal adenocarcinoma (PDA), NLRP3 drives IL-10 dependent expansion of immune-suppressive macrophages and promotes tumors (38) (**Table 1**).

**Table 1:** Inflammasomes and their pro-tumorigenic and anti-tumorigenic role in different Cancers.

Pro-tumorigenic roles of inflammasome		
Inflammasome	Cancer	Mechanism (s)
NLRP3	CRC	EMT (35)
	BC	Autocrine IL-1β secretion and EMT (36)
	Lung cancer	Activation of NLRP3 by TRIM59 (37)
	OSCC	IL-1β secretion and EMT (32)
	PDA	IL-10 dependent activation of immunosuppressive macrophages (38)
AIM2	CRC	Blocking cell cycle in G2/M phase and promoting invasion (50)
	OSCC	STAT1/NF-κB (56)
NLRC4	Prostate cancer	IL−1β and IL−18 (70)
	Glioma	Tim-3/Gal-9 (71)
Anti-tumorigenic roles of inflammasome		
NLRP3	Colitis-associated cancer	PYCARD and caspase-1 (21)
	HCC	Absences or low expression of NLRP3 (39)
	LUAD	Activation and proliferation of T cells (42)
AIM2	CRC	1. Limiting the intestinal stem cell proliferation and controlling gut microbiome (52) (53), 2. Inhibition of DNA-PK and Akt pathway (54)
	HCC	Inhibition of mTOR-S6K1 pathway (55)
NLRC4	Melanoma	Activation of inflammatory signaling in macrophages and to enhance CD4^+^ and CD8^+^ T cells (68)
	CRC	Immune cell infiltration (69)

CRC, Colorectal cancer; BC, Breast cancer; OSCC, Oral squamous cell carcinoma; PDA, Pancreatic ductal adenocarcinoma; HCC, Hepatocellular carcinoma; LUAD, Lung adenocarcinoma.

Several studies have also shown the anti-tumorigenic effects of NLRP3 in various cancer types including pancreatic carcinoma and hepatic cancer. For example, the NLRP3 inflammasome has been shown identified as a negative regulator of tumorigenesis in colitis-associated cancer. Mice deficient in PYCARD, caspase-1, and NLRP3 showed worsened inflammation, increased tumor burden, and decreased levels of IL-1β and IL-18 (21). Similarly, in hepatocellular carcinoma (HCC), NLRP3 plays a protective role, as the expression of NLRP3 inflammasome partner proteins is either completely absent or significantly low in hepatic cancer cells (39) (**Table 1** **).**

Furthermore, Han et al. (2021), showed that radiation-induced NLRP3 inflammasome activation promotes anti-tumor immunity. This occurs through the IL-1R mediated activation of dendritic cell and T cell responses in mice (40). Additionally, a study by Fan et al. (2021) showed that *Akkermansia muciniphila* (A. muciniphila), a bacterium naturally found in the human gut, triggers TLR2 dependent NF-kB/NLRP3 pathways in M1-like macrophages to inhibit tumor growth (41). Combination of radiotherapy and NLRP3 agonist in wild type and anti-PD1 resistant murine-implanted lung adenocarcinoma (LUAD) models have also shown to boost immune response via activation and proliferation of T cells and thus shows improved anti-tumor effect (42) (**Table 1** **).** NLRP3 inflammasome induced by radiation can also lead to tissue damage. Chest radiation therapy is important in case of thoracic and breast malignancies but leads to radiation induced lung injury (RILI). Rao et al. (2023) showed that NLRP3 inflammasome activation plays a major role in acceleration of RILI by release of IL-1β that promotes fibroblast migration, proliferation and activation. Repression of both NLRP3 and IL-1β had shown a reversed effect and decreased RILI (43).

## AIM2 inflammasome activation and its role in cancer progression

Absent in melanoma 2 (AIM2) was initially identified as a tumor suppressor gene and is predominantly expressed in epithelial cells and macrophages, playing a crucial role in immune surveillance and inflammation. When double-stranded DNA (dsDNA) is released into the cytosol from host cells or from pathogens such as viruses, bacteria or fungi during infection, AIM2 recognizes the dsDNA, triggering the formation of an inflammasome (33). The activation process of the AIM2 inflammasome complex is similar to that of the NLRP3 inflammasome, involving the recruitment of ASC (apoptosis-associated speck-like protein containing a CARD) and procaspase-1, ultimately resulting in the activation of caspase-1. Upon activation, the inflammasome signaling cascade promotes the cleavage of proinflammatory cytokines pro-IL-1β and pro-IL-18, leading to release of mature IL-1β and IL-18 inducing inflammation. Additionally, activated caspase-1 cleaves GSDMD, inducing pyroptotic cell death (**Figure 1**) (33, 44–47). These proinflammatory cytokines not only trigger the innate immune response but also protect from intestinal inflammation and colitis-associated colon cancer. The presence of a microsatellite site in the AIM2 gene renders it susceptible to frequent mutations, implicated in CRC (48, 49), an inflammasome independent function.

AIM2 also plays a dual role in cancer progression, functioning through both inflammasome-dependent and independent mechanisms. It is linked to promotion of colon (50) and NSCLC (51). As mentioned in **Table 1**, Man et al. (2015) and Rommereim et al. (2015) investigated AIM2 involvement in inhibition of colon cancer by limiting the intestinal stem cell proliferation and controlling gut microbiome (52, 53). A study by Pasto et al. (2009) showed that AIM2 inflammasomes arrest cells in the G2/M phases of the cell cycle, blocking progression of cell cycle and promoting invasion of CRC (**Table 1**) (50). Furthermore, AIM2’s inflammasome-independent role in inhibiting colon cancer is mediated by the suppression of DNA-dependent protein kinase (DNA-PK) activation and Akt signaling pathways (54). In HCC, AIM2 controls tumor growth by inhibiting key regulatory protein kinases, particularly the mTOR-S6K1 pathway as described in **Table 1** (55).

Radiation therapy is commonly used to eliminate cancerous cells in affected tissues, but it can also cause cellular and mitochondrial damage in tumors, leading to the release of nuclear and mitochondrial DNA (mtDNA) into the cytosol. This released DNA acts as a potent trigger for innate immune sensing by the AIM2 inflammasome and cGAS-STING pathways, resulting in the activation of the type I interferon and proinflammatory cytokines production. These pathways play a crucial role in the immunogenic effects of radiation by promoting local inflammation, immune cell recruitment, and tumor clearance. Han et al. (2021) demonstrated that radiation activates AIM2 inflammasome, which contributes to anti-tumor effects through inflammatory cytokines like IL-1 (40). AIM2 has also been implicated in promoting radiation resistance in oral squamous cell carcinoma (OSCC) through the STAT1/NF-κB pathway (56). Overall, AIM2 plays a significant role in tumor progression and immune regulation, making it a key target for therapeutic intervention. The cGAS-STING signaling pathway exerts anti-tumor effects in cancer cells through both cell-autonomous and non-cell-autonomous actions. Cell-autonomously, activation of STING within tumor cells can promote apoptosis and reinforce oncogene-induced senescence (OIS) by inducing the secretion of senescence-associated secretory phenotype (SASP) factors, thereby limiting tumor cell proliferation (57, 58). Non-cell-autonomously, STING activation leads to the production of the type I interferons and other pro-inflammatory cytokines, which facilitate cross-talk between tumor cells and immune cells in the tumor microenvironment, enhancing antigen presentation and promoting robust antitumor immune response (59, 60).

## NLRC4 inflammasome activation and its role in cancer progression

The Nod-like receptor C4 (NLRC4), initially referred to as *Ipaf*, was identified as an activator of procaspase-1 (61). NLRC4 plays a crucial role in the bacterial innate immune response through inflammasome activation. NLRC4 interacts with NLR apoptosis inhibitory proteins (NIAP), which act as receptors for bacterial ligands like flagellin and proteins of bacterial type III secretion systems (62, 63). Karki et al. (2018) demonstrated that Interferon Regulatory Factor 8 (IRF8) regulates the transcription of *Niap* genes for the optimal activation of NLRC4 upon bacterial infections (64). Upon sensing these bacterial ligands, NIAP interacts with NLRC4 to facilitate the assembly of the NIAP-NLRC4 inflammasome (62–65). The NIAP-NLRC4 oligomerizes and recruits the adaptor protein ASC, leading to cleavage of pro-caspase-1 into active caspase-1. Caspase-1 then cleaves proinflammatory cytokines, like pro-IL-1β and pro-IL-18, into their active forms and activates the pore-forming protein gasdermin D, which triggers inflammation and pyroptotic cell death (**Figure 1**) (9, 66). A recent study demonstrated that ionizing radiation stimulates the production of IL-1β in macrophages through the p38-MAPK-NLRC4 signaling pathway. Silencing NLRC4 with RNA interference reduced the radiation-induced increase in IL-1β production (67).

Similar to AIM2 and NLRP3, NLRC4 is implicated in various types of cancer. In the melanoma *Nlrc4^-/-^* mice model, it was identified that NLRC4 inhibits tumor growth. NLRC4 was associated with the activation of inflammatory signaling in macrophages and to enhance the production of IFN-γ by CD4^+^ and CD8^+^ T cells to inhibit melanoma progression (68) (**Table 1**). In case of CRC tissues and cell lines reduced levels of NLRC4 and CASP1 were observed. Decreased expression of NLRC4 and CASP1 was associated with poor survival, lymph node metastasis, and affected immune cell infiltration (69) (**Table 1** **).**

The inflammatory microenvironment driven by NLRC4 inflammasomes can promote malignancy in various tissues, including prostate and glioma. In prostate cancer, elevated NLRC4 expression has been linked to an increased risk of tumor progression might be because of secretion of IL−1β and IL−18 (**Table 1** **) (** 70). Similarly, higher NLRC4 expression is observed in glioma tissues, where it plays a role in tumor progression (71, 72). Tim3/Gal9 activates expression of NLRC4 and caspase 1 via FYN-JAK1-ZNF384 and promotes malignancy in glioma as indicated in **Table 1**. NLRC4 levels may serve as a diagnostic biomarker for both prostate and glioma cancers. Additionally, NLRC4 could be a potential therapeutic approach for various cancer treatments.

## Conclusions and future perspectives

Inflammasomes such as NLRP3, AIM2, and NLRC4, are emerging as crucial regulators of cancer-related inflammation, playing diverse roles in tumor initiation, progression, and immune surveillance. These innate immune sensors detect cellular stress signals and trigger inflammatory responses through the maturation of IL-1β and IL-18 and the induction of pyroptosis. While inflammasome activation can derive antitumor immunity by increasing immune cell infiltration and clearing tumor cells, it can also promote tumor progression by creating a chronic inflammatory microenvironment, immunosuppression, and metastasis, depending on the tumor type and context.

NLRP3 has been implicated in both pro-tumorigenic and antitumor functions, with evidence supporting its role in promoting tumorigenesis through IL-1β-mediated inflammation, while also contributing to immune-mediated tumor control. AIM2 classically known for its role in sensing cytosolic DNA, can induce inflammasome activation and tumor cell pyroptosis, yet it may also suppress tumor progression through non-inflammasome functions such as DNA damage repair regulation. NLRC4, although less extensively studied in cancer, has been shown both tumor-suppressive and tumor promoting roles, particularly affecting myeloid cell function and cytokines production.

The roles of other inflammatory sensor proteins, such as NOD-like receptors (including NLRP1 (73), NLRP2 (74), NLRP6 (75), NLRP7 (76), and NLRP12 (77), AIM2-like receptors (ALRs) like IFI16 (78), Caspase-11 (caspase 4/5 in humans) inflammasome (79), Pyrin inflammasomes (80) and TRIM proteins (81) in cancer development, are not fully understood. Therefore, understanding the precise molecular pathways through which these inflammatory sensors control specific types of cancer is crucial for developing targeted therapeutic strategies. Future research should also focus on identifying context-specific regulators of inflammasome activity and exploring the potential of inflammasome-targeted therapies in cancer treatment. Combinatorial approaches that combine inflammasome modulation with immunotherapy or radiotherapy may offer new avenues for effective cancer management.
